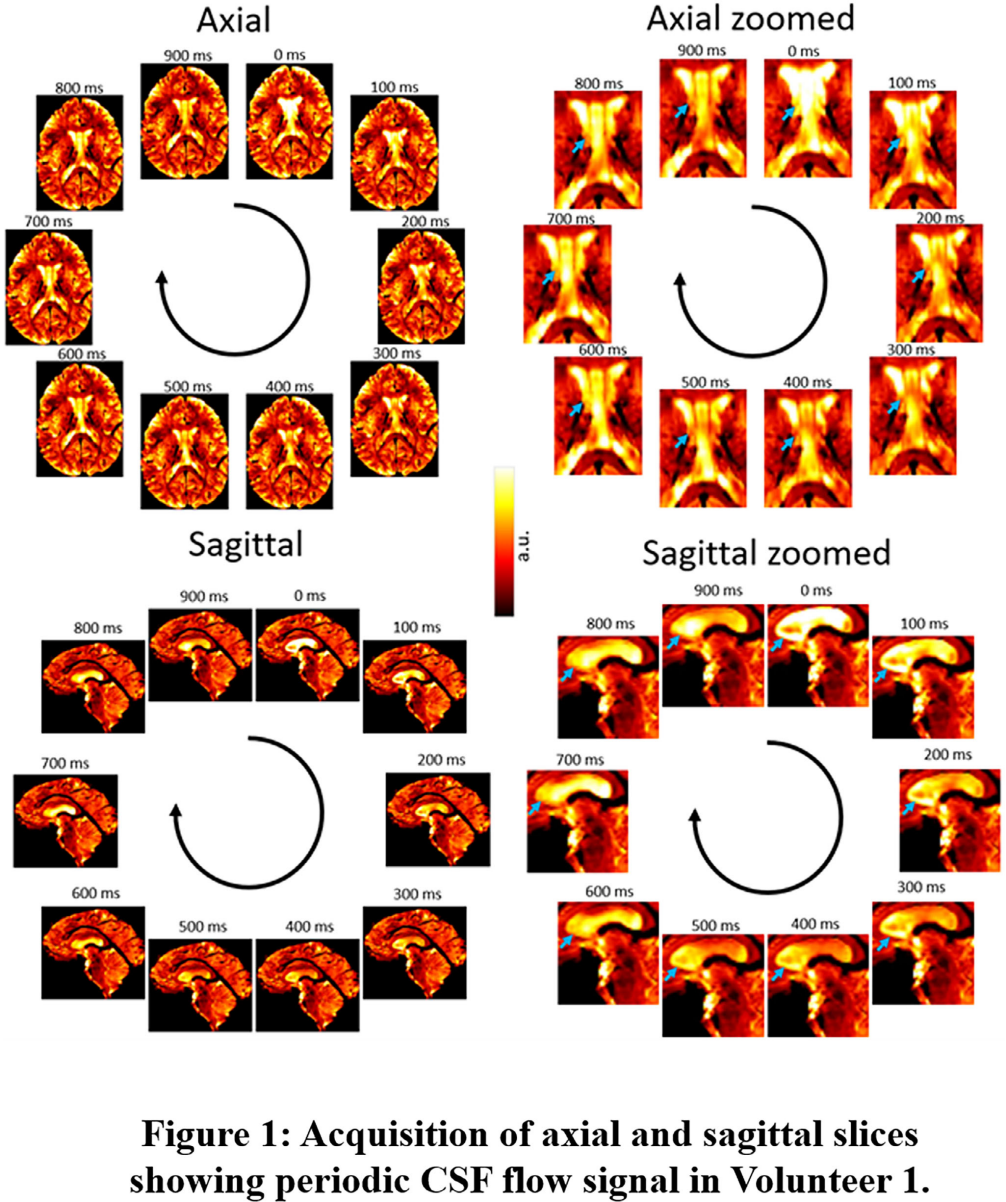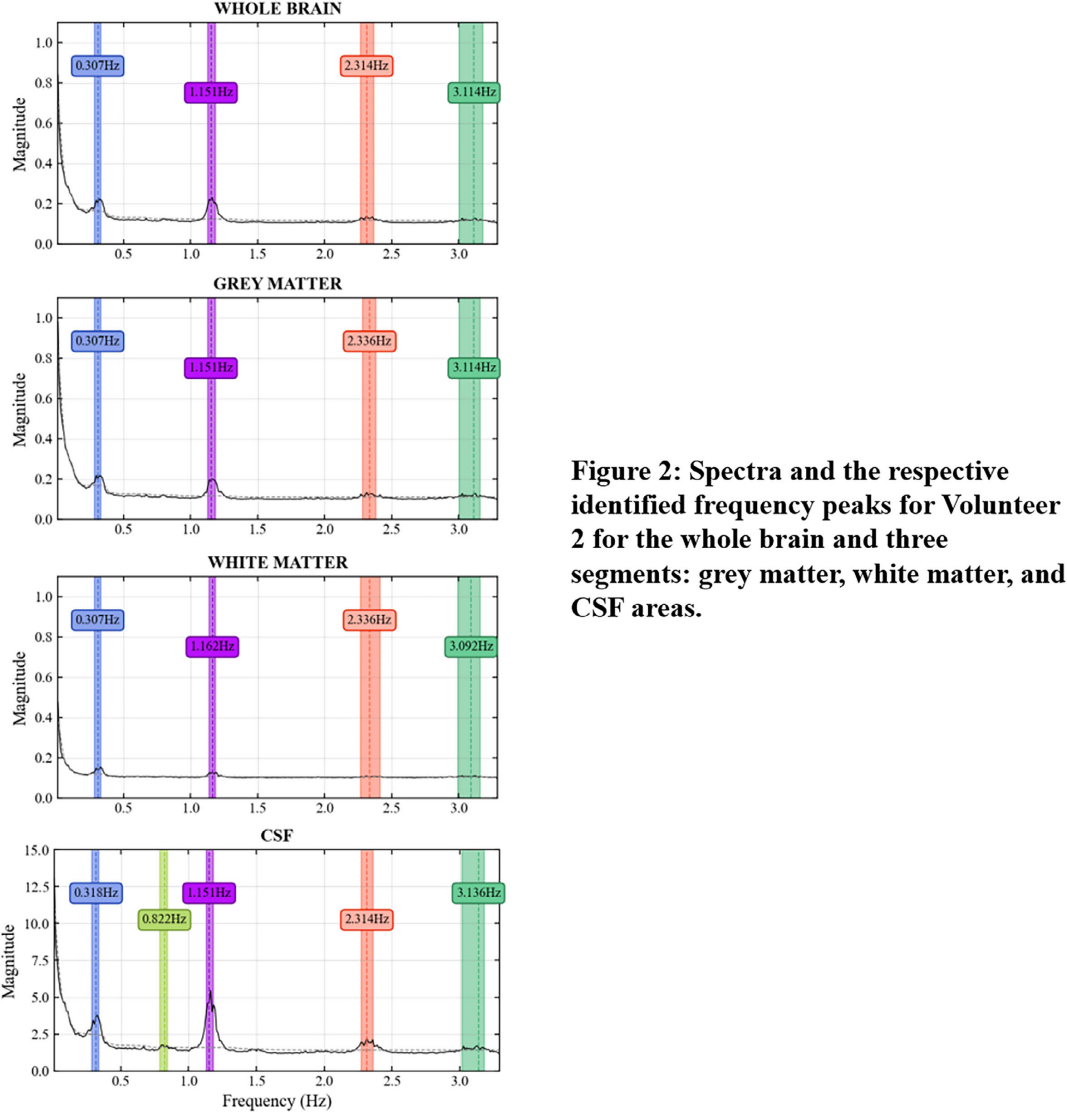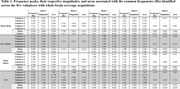# Characterization of pulsations in the brain and cerebrospinal fluid using ultra‐high field magnetic resonance imaging

**DOI:** 10.1002/alz.092958

**Published:** 2025-01-09

**Authors:** Bruno de Almeida, Tiago Amaro Martins, Minjie Wu, Kristine A Wilckens, Davneet S Minhas, James W Ibinson, Howard J Aizenstein, Tales Santini, Tamer S Ibrahim

**Affiliations:** ^1^ University of Pittsburgh, Pittsburgh, PA USA

## Abstract

**Background:**

Brain fluid flow plays a crucial role in maintaining brain health by eliminating potentially harmful waste products like amyloid‐beta and tau [1‐2]. This process is potentially facilitated by pulsations in the perivascular space, influenced by the neurovascular unit and autonomic nervous system, which may vary in brain diseases such as Alzheimer’s disease (AD) [3‐4]. Using a 7 Tesla MRI scanner and ultrafast echo‐planar imaging (EPI), we developed a non‐invasive neuroimaging methodology to characterize the in‐vivo frequency and amplitude responses of pulsations of cerebrospinal fluid (CSF) flow.

**Method:**

Brain images from six volunteers were acquired using a 7T MRI Scanner and an in‐house developed 16‐channel Tic‐Tac‐Toe transmit array with a 32‐channel receive coil. The human‐connectome EPI multiband MR sequence was optimized to present a real‐time visualization of the CSF flow in the brain. For the first volunteer, two axial and one sagittal slices were obtained, while whole‐brain coverage was acquired for the remaining volunteers, where the acquisition was divided into 19 slabs, each consisting of 3 axial slices. A spectral analysis based on Fast Fourier Transform (FFT) was conducted for the whole brain and grey matter, white matter, and CSF regions.

**Result:**

Figure 1 presents the processed axial and sagittal slices acquired from the first volunteer, demonstrating the presence of a periodic variation of the CSF signal. Figure 2 presents the identification of peaks in the spectrum acquisitions of whole‐brain segments for one volunteer, which exhibited similar frequencies, magnitudes and bandwidths among different volunteers. Quantitative results of this analysis are summarized in Table 1.

**Conclusion:**

This project developed a real‐time visualization of CSF pulsations based on a non‐invasive ultrafast EPI obtained with a 7T MRI scanner. The identification of similar frequency peaks (around 0.3, 0.8, 1.1, 2.3 and 3.1 Hz) and magnitudes among healthy volunteers suggests its potential as a feasible biomarker. Future work will examine this technique’s potential to identify changes in brain fluid dynamics associated with AD progression and pathology.

**Funding**: NIH R01AG063525, R01MH111265, University of Pittsburgh CRC's RRID:SCR_022735.

**References**:

Nedergaard, 2013

Xie et al., 2013

Peng et al., 2016

Ramanathan et al., 2015